# Interrelation between Tween and the membrane properties and high pressure tolerance of *Lactobacillus plantarum*

**DOI:** 10.1186/s12866-018-1203-y

**Published:** 2018-07-13

**Authors:** Dominik Reitermayer, Thomas A. Kafka, Christian A. Lenz, Rudi F. Vogel

**Affiliations:** Technische Universität München, Freising, Germany

**Keywords:** HHP, High hydrostatic pressure inactivation, Pasteurization, Polysorbate 80, Tween, Emulsifier, Food additive E 433, Lactobacilli, Lactobacillus plantarum, Food spoilage, Cytoplasmic membrane, Fatty acids, Oleic acid

## Abstract

Tween® 80 is a frequently used supplement of media for the cultivation of lactic acid bacteria. We investigated its effect on the cell physiology and stress tolerance of *Lactobacillus* (*L.*) *plantarum*. Data on the transcriptomic response to Tween 80 supplementation and its effects on cellular fatty acid profiles and growth characteristics are compared with data characterizing the effect of Tween 80, other Tween types and free fatty acids on the high hydrostatic pressure (HHP) tolerance of *L. plantarum* strain TMW 1.708. These include effects on cell viability, sub-lethal injury, metabolic activity, protein release and propidium iodide uptake. Tween 80 caused the downregulation of fatty acid biosynthesis and an increase in oleic acid and cyclopropane fatty acid levels in the cell membrane. Tween 20, Tween 80 and free oleic acid, but not Tween 40, Tween 60 and other free fatty acids, conferred resistance against HHP. Tween 80 diminished pressure-induced loss of metabolic activity, protein release and uptake of propidium iodide. However, loss of cell viability exceeded by far membrane permeabilization, suggesting that membrane permeabilization, which has frequently been postulated as a major factor in HHP inactivation of microbes, is not necessarily required for HHP-induced cell death of *Lactobacillus plantarum*.

**Electronic supplementary material:**

The online version of this article (10.1186/s12866-018-1203-y) contains supplementary material, which is available to authorized users.

## Background

Polyoxyethylene (20) sorbitan monooleate, frequently referred to as polysorbate 80 or its registered trade name, Tween® 80, is a non-ionic surfactant and emulsifier often used in foods (EU food additive number E 433), cosmetics and medications for parenteral administration. Furthermore, Tween 80 is widely used as an additive in microbiological growth media, providing bacterial cells (e.g. from *Lactobacillus* species) with exogenous oleic acid [1], which constitutes the lipophilic group of Tween 80. Additionally, oleic acid is one of the most abundant fatty acids in many plant and animal fats and is therefore present in a variety of food products.

Consequentially, bacterial cells from food-associated species are likely to get in contact either with Tween 80 itself or its fatty acid group during enrichment in cultivation medium or in an actual food product. A prominent example for food-associated bacterial species is *Lactobacillus* (*L.*) *plantarum*, which is widely used in food and feed fermentations, has been claimed to have probiotic traits, but has also been associated with food spoilage [2–4].

Various effects of Tween 80 on vegetative cells have been described. It can enhance growth of lactobacilli [5–8] and protect cells against adverse environmental conditions, including acidity [9, 10], bile salts [11], freeze-drying [12] and nutrient depletion [13]. These effects are potentially related to the fact that the oleic acid moiety of Tween 80 can be incorporated into the cell membrane, which affects cell membrane properties [6, 7, 9]. Furthermore, it was shown that exogenous fatty acids can be directly incorporated into the cells, and that oleic acid supplementation represses fatty acid synthesis in *L. plantarum*, where reduced production of responsible enzymes could play a role [14, 15]. However, the exact physiological mechanism underlying growth enhancement and stress protection by Tween 80 is not yet completely clear.

Additionally, there is a large gap of knowledge regarding possible effects of Tween 80 or fatty acids on the resistance of vegetative cells to novel food preservation technologies. These include high hydrostatic pressure (HHP) processing, which has the potential to inactivate vegetative microorganisms while retaining valuable food attributes, such as flavors, texture, colors and vitamins.

Previously obtained data from our group shows that the presence of fat per se during HHP treatment does not protect *L. plantarum* cells against HHP-mediated inactivation [16]. However, it is known that many food environments can protect microorganisms from efficient HHP inactivation, and the effect of pre-conditioning in fat- or fatty acid-containing environments before HHP treatments has not been investigated so far. In this context even more importantly, HHP appears to be a suitable physical parameter to probe the impact of cell membrane composition on the structure and function of bacterial cell membranes as determined by their growth and survival.

Results from several studies imply that the cytoplasmic membrane is one of the main cellular target structures for HHP inactivation of vegetative cells, i.e. that (i) the membrane undergoes phase transition from the liquid-crystalline to the gel phase accompanied by permeabilization [17, 18], (ii) the function of membrane-bound enzymes is impaired by pressure-induced phase transition [19], (iii) membrane properties, including fluidity, phase transition temperature and fatty acid composition strongly influence the sensitivity of bacteria to HHP treatments [19, 20], and (iv) barophilic bacteria adjust the fatty acid composition of their membranes according to growth pressure in order to maintain proper fluidity and functionality [21]. Together with the fact that cultivation in the presence of Tween 80 can alter membrane composition, it can be hypothesized that Tween 80 can directly influence HHP sensitivity.

A better understanding of the mechanisms of the cellular response of lactobacilli to Tween 80 could enable a more efficient use of this substance as well as a more target-oriented study design and development of growth and fermentation conditions. On the other hand, a deeper knowledge of the processes causing stress resistance would facilitate the development of strategies to avoid or reduce the stress-protecting effect of Tween 80 or oleic acid, thus increasing the efficiency of HHP as food preservation method.

Thus, we aimed to gain deeper insight into the mechanism underlying the growth-enhancing and protective effect of Tween 80 on lactobacilli. For this purpose, we tested whether there exists a link between the supplementation of the growth medium with Tween 80, the membrane fatty acid composition and the high pressure sensitivity of *L. plantarum*. This was done by (i) the determination of the transcriptomic response and changes in the membrane fatty acid profiles upon cultivation in the presence of Tween 80 and (ii) the assessment of the effect of Tween 80 on cell viability, sub-lethal injury, metabolic activity and membrane damage after HHP treatments.

## Methods

### Bacteria and culture conditions

*Lactobacillus plantarum* strain TMW 1.708 was stored at − 80 °C in a 1:1 mixture of 80% glycerol and modified MRS (mMRS) medium (10 g L^− 1^ peptone from casein, 5 g L^− 1^ meat extract, 5 g L^− 1^ yeast extract, 4 g L^− 1^ KH_2_PO_4_, 2.6 g L^− 1^ K_2_HPO_4_ * 3 H_2_O, 3 g L^− 1^ NH_4_Cl, 0.5 g L^− 1^ cystein-HCl, 1 g L^− 1^ Tween 80, 7.5 g L^− 1^ glucose, 7.5 g L^− 1^ fructose, 0.1 g L^− 1^ MgSO_4_ * 7 H_2_O, 0.05 g L^− 1^ MnSO_4_ * 4 H_2_O, pH 6.2) [22]. Glucose and fructose were autoclaved separately. A 1000 × stock solution of MgSO_4_ * 7 H_2_O and MnSO_4_ * 4 H_2_O was filter-sterilized (0.2 μm pore size, Sarstedt, Nürnbrecht, Germany). Both solutions were added to the medium after autoclaving. For activation of the strain, a loopful of the cryo culture was transferred to mMRS medium from which Tween 80 was omitted (mMRS-). The culture was incubated at 30 °C overnight. The overnight culture was used as inoculum in the experiments.

### mMRS medium containing different tween types

*Lactobacillus plantarum* was either grown in mMRS- or in mMRS containing 1 g L^− 1^ Tween 20 (mMRST20), Tween 40 (mMRST40), Tween 60 (mMRST60) or Tween 80 (mMRST80). All Tween types were purchased from Merck (Darmstadt, Germany). The different Tween types were solubilized in mMRS- at a concentration of 10 g L^− 1^, filter-sterilized (0.2 μm pore size, Sarstedt, Nürnbrecht, Germany), and finally added to mMRS- to give a concentration of 1 g L^− 1^.

### mMRS medium containing free fatty acids

Free fatty acids, i.e. stearic acid (Merck Schuchard, Hohenbrunn, Germany), oleic acid (Merck Darmstadt, Germany), linoleic acid (Sigma, Steinheim, Germany), linolenic acid (Sigma, Steinheim Germany), palmitic acid (Merck Schuchard, Hohenbrunn, Germany), palmitoleic acid (Fluka Sigma-Aldrich, St Louis, MO, USA), myristic acid (Merck Schuchard, Hohenbrunn, Germany), or lauric acid (Merck, Darmstadt, Germany) were solubilized in 95% ethanol to give a concentration of 50 mM. Either one of these stock solutions was added 1:1000 to mMRS- to obtain a final fatty acid concentration of 50 μM.

### Transcriptomic analysis

In order to investigate the effect of Tween 80 on the metabolism of *L. plantarum* TMW 1.708, the transcriptomic response to medium supplementation with Tween 80 was assessed. For this, sterile mMRS- was inoculated (1% (*v*/v)) with an overnight culture of TMW 1.708 and incubated at 30 °C for 24 h. Another 9 mL mMRS- were inoculated with 1% (v/v) of this 24 h culture and grown at 30 °C for 4 h, followed by the addition of 1 mL of either mMRS- or mMRS containing 10 g L^− 1^ Tween 80. Transcription was stopped after 0.5 h incubation at 30 °C by the addition of RNAprotect Bacteria Reagent (Qiagen, Hilden, Germany) to the culture. Thereafter, mRNA was isolated and purified using the RNeasy mini kit (Qiagen, Hilden, Germany) according to the manufacturer’s protocol. Final RNA concentration was determined via light absorption spectrometry using a NanoDrop™ device (Wilmington, DE, USA). Samples were then sent to GATC Biotech (Constance, Germany) for RNA sequencing. Sequencing data were analyzed using Rockhopper software on the basis of the genome of *L. plantarum* TMW 1.708 (Biosample: SAMN05805046 [23]) [24, 25]. Genes with corrected *p*-values lower than 0.05 were considered to show significantly different expression levels. The 2.5% quantiles with the greatest increase and decrease in expression level were considered for further investigation.

### Examination of growth parameters

In order to evaluate the effect of Tween 80 on characteristic growth parameters of *L. plantarum* TMW 1.708 growth curves were recorded by OD_600_ measurement. In addition to Tween 80, three additional Tween types were included in the study, namely Tween 20, Tween 40 and Tween 60, which are characterized by lauric acid, palmitic acid and stearic acid as their specific fatty acid moiety, respectively. For this, mMRS-, mMRST20, mMRST40, mMRST60 and mMRST80 were inoculated (1% (*v*/v)) with a 24 h culture of TMW 1.708 in mMRS-. An aliquot of each inoculated medium (150 μL per well) was then transferred to a 96-well plate (sterile, transparent, F-bottom, Sarstedt, Nürnbrecht, Germany). Each well was covered with 50 μL sterile paraffin oil to avoid evaporation. The plate was incubated at 30 °C in a FLUOStar Omega plate reader (BMG Labtech, Ortenberg, Germany) for 30 h. The optical density at 600 nm (OD_600_) was measured every 30 min following agitation with 200 rpm for 30 s.

### Determination of membrane fatty acid composition

For the determination of cellular fatty acid profiles, sterile mMRS- was inoculated with 1% (*v*/v) of an overnight culture of TMW 1.708 and incubated at 30 °C for 24 h. Fresh mMRS- or mMRST20/40/60/80 was inoculated (1% (v/v)) with the 24 h culture and incubated at 30 °C for another 24 h to give a cell density of approximately 10^9^ colony forming units (cfu) mL^− 1^. Cells were harvested by centrifugation (5000×g, 25 °C, 5 min), washed three times in ¼-strength Ringer’s solution and finally freeze-dried using a FreeZone Plus 2.5 L freeze dry system (Labonco, Kansas City, MO, USA). The lyophilisate was stored under N_2_ atmosphere. Fatty acid analyses were carried out by the Identification Service of the DSMZ (Braunschweig, Germany).

### HHP treatment

For HHP treatments, cells grown in the respective medium at 30 °C for 24 h were centrifuged (5.000×g, 25 °C, 5 min), washed once in imidazole/phosphate buffer (IPB, 0.1 g L^− 1^ KH_2_PO_4_, 4.45 g L^− 1^ NA_2_HPO_4_ * 2 H_2_O, 1.7 g L^− 1^ imidazole, pH 7.0) and, finally, resuspended in IPB to give 10^7^ –10^8^ cfu mL^− 1^ for inactivation experiments, and 10^8^ –10^9^ cfu mL^− 1^ for experiments examining metabolic activity, protein release and membrane permeability to ensure a sufficient signal strength during measurements. An aliquot of 600 μL of the cell suspension was transferred to cryovails (0.5 mL Nunc CryoTube™ Vials, internal thread, Thermo Fisher Scientific, Waltham, MA, USA). Care was taken to avoid the inclusion of air bubbles. Samples were pressurized in two parallel linked 7 mL pressure vessels equipped with thermostating jackets (high pressure unit TMW-RB, described earlier [26, 27]). A mixture of 70% polyethylene glycol 400 (Roth, Karlsruhe, Germany) and 30% deionized water was used as pressure-transmitting fluid. Vessel temperature was held constant at 25 °C (FC 600; JULABO, Seelbach, Germany). Prior to starting the pressure ramp, samples were incubated for 5 min in the pressure vessel to reach the desired starting temperature. Compression and decompression rates were kept constant at 200 MPa min^− 1^. Target pressure/holding time combinations were chosen upon their capability of inactivating a significant portion of *L. plantarum* cells within a population and are indicated individually for each experiment in the results section.

### Effect of HHP on cell viability

Pressurized and unpressurized control samples were diluted in isotonic tryptone solution supplemented with Antifoam B (145 mM NaCl, 14 g L^− 1^ tryptone, 0.01% (*v*/v) Antifoam B Emulsion). Serial dilutions were spread-plated on mMRS supplemented with 15 g L^− 1^ agar using glass beads. For the determination of sub-lethal injury, the agar was supplemented with 7% NaCl (*w*/*v*), and the maximum non-inhibitory concentration according to literature [28] was determined for *L. plantarum* TMW 1.708 in a separate experiment (data not shown). The plates were incubated for 72 h at 30 °C to allow for cell recovery and colony formation. High pressure inactivation experiments were performed at least in independent triplicate.

### Effect of HHP on metabolic activity

To determine the metabolic activity after HHP treatment, a stock solution (70 mM) of resazurin-Na salt (Serva, Heidelberg, Germany) in IPB was prepared, diluted to 1 μM in IPB containing 15 g L^− 1^ glucose and 15 g L^− 1^ fructose, and filter-sterilized (0.2 μm, Sarstedt, Nürnbrecht, Germany), giving the resazurin working solution used. To measure metabolic activity, 100 μL resazurin working solution were mixed with 100 μL pressure-treated or untreated cell suspension on white 96-well microtiter plates (F-bottom, Nunc, Thermo Fisher Scientific, Waltham, MA, USA), and fluorescence intensity (ex/em: 544/590 nm) was measured during incubation at 30 °C in an Omega FLUOStar microplate reader (BMG Labtech, Ortenberg, Germany) every 120 s for a period of 30 min. The assay is based on the fact that the blue dye resazurin is reduced by cellular enzymes to red fluorescent resorufin. The reaction runs faster the more metabolically active cells are present in a sample, so that the metabolic activity can be determined from the slope of fluorescence increase. The increase in fluorescence intensity over time expressed as percent of the value of untreated cells was used to estimate HHP-induced changes in the metabolic activity of *L. plantarum* cells.

### Effect of HHP on protein release

For the determination of protein release upon high pressure treatment, an aliquot of 500 μL of a pressure-treated sample (10^8^–10^9^ cfu mL^− 1^) was transferred to a sterile reaction tube and centrifuged (2800×g, 5 °C, 15 min). The supernatant was filter-sterilized (0,2 μM pore size, Sarstedt, Nürnbrecht, Germany) and protein concentration was measured on black 96-well microtiter plates (F-bottom, Greiner bio-one, Frickenhausen, Germany) using the Pierce™ Coomassie Plus (Bradford) assay kit (ThermoFisher, Waltham, MA, USA) according to the micro MTP protocol provided by the manufacturer. The samples were incubated at 25 °C in the dark for 10 min and absorbance at 595 nm was measured in a FLUOStar Omega plate reader (BMG LABTECH GmbH, Ortenberg, Germany). Bovine serum albumin provided with the kit was used to establish a standard curve for protein concentrations between 0 and 25 μg mL^− 1^.

### Effect of HHP on membrane permeability

Membrane permeability was determined using propidium iodide (PI) fluorescence, as described earlier [29]. For the determination of transient membrane permeability, cell suspensions (10^8^–10^9^ cfu mL^− 1^) in IPB were mixed with PI (Invitrogen, Thermo Fisher Scientific, Waltham, MA, USA) to a final concentration of 3 μM before HHP treatment. After pressure release, samples were washed twice in IPB (10,000×g, 5 min, 25 °C) and finally resuspended in twice the original volume of IPB. Permanent membrane permeability was determined by PI staining immediately after HHP treatment followed by incubation for at least 15 min in the dark and subsequent purification as described above. Suspensions of untreated and heat-inactivated (100 °C, 15 min) cells were mixed with PI in parallel to the pressure-treated samples and were used as negative and positive controls, representing populations without membrane damage and thus minimum fluorescence intensity or with completely destroyed membranes leading to the maximum achievable fluorescence signal, respectively. Membrane permeability was determined via the measurement of the fluorescence intensity (excitation/emission: 485/620 nm) on black 96-well plates (F-bottom, Greiner bio-one, Frickenhausen, Germany) using a FLUOStar Omega microplate reader (BMG Labtech, Ortenberg, Germany). For normalization purposes, OD_600_ was measured in parallel (U-bottom, non-sterile, Sarstedt, Nürnbrecht, Germany). For microscopic analysis, samples were treated as explained above with the exception that staining was performed using the LIVE/DEAD BacLight Bacterial Viability kit (Thermo Fisher Scientific, Waltham, MA, USA) containing SYTO®9 and PI. The final concentration of each dye in the sample was 3 μM. An aliquot of 5 μL stained sample was spread on a glass microscope slide (Roth, Karlsruhe, Germany), covered with a coverslip and examined under an Axiostar plus microscope (Carl Zeiss Microscopy GmbH, Jena, Germany) equipped with an epifluorescence unit. Stained cells were visualized using epifluorescence light with the appropriate filters (SYTO®9: Excitation BP 475/40, Emission BP 530/50; Propidium Iodide: Excitation BP 546/12, Emission LP 590). In all cases, a 100× objective was used, giving a total magnification of 1000-fold. Images were captured with a 1.388 × 1.038 pixel RGB camera (AxioCam ICc1) and processed with AxioVS40 V 4.8.2.0 software (Carl Zeiss MicroImaging, Jena, Germany).

### Data analysis

To determine the significance of the differences between measured values, a Student’s t-test was used for the comparison of two and the analysis of variance (ANOVA) for the comparison of more values. Where ANOVA values were significant, ANOVA analyses were followed by a post-hoc Student-Newman-Keuls test for paired comparison. Statistical analyses were performed using the software Sigmaplot. Results were considered significantly different when *p*-values were < 0.05.

## Results

### Transcriptomic analysis

Additional file 1: Table S1 provides an overview of the lower (A) and upper (B) 2.5% quantiles of genes for which significant changes in their expression levels were detected in response to medium supplementation with Tween 80. By far the most pronounced effect was observed with central components of the fatty acid synthesis pathway, which were strongly downregulated upon the addition of Tween 80 (Additional file 1: Table S1a). Moreover, genes responsible for the transmembrane transport of oligopeptides (*oppC*) and potassium (*kup1/2*) as well as gadB glutamate decarboxylase, which catalyzes the formation of 4-aminobutanoate and CO_2_ from glutamate, were downregulated in mMRST80. In contrast, the expression of genes implicated in lysine uptake (*lysP*) and the uptake (*glnQ3*, *glnPH2*) and synthesis (*glnA*) of glutamine was enhanced upon the addition of Tween 80. Upregulation was observed also for genes involved in manganese (*mntH3*, predicted protein) and phosphate (*pstB1*) uptake, transmembrane diffusion of uncharged solutes such as glycerol, urea and lactic acid (*glpF4*), ingestion of N-acetylglucosamine (*pts18CBA*) as well as pyrimidine metabolism. The latter include (i) a predicted transmembrane permease for uracil uptake, pyrP [30], (ii) orothate phosphoribosyltransferase pyrE, which is involved in the formation of uridine monophosphate (UMP) from orothate [31], (iii) *pyrG* coding for cytidine triphosphate (CTP) synthase, which catalyzes the synthesis of CTP and glutamate from uridine triphosphate (UTP) and glutamine, and (iv) the transcriptional regulator of the biosynthetic pyrimidine *pyrR1-B-C-Aa1-Ab1-D-F-E* operon, pyrR1 [32]. This points towards increased metabolic activity in the presence of Tween 80. Furthermore, two genes for predicted N-acetyltransferases, part of the *cps4* gene cluster including genes with predicted glycosyltransferase (*cps4G*, *cps4I*) polysaccharide polymerase (*csp4H*) and flippase (*csp4J*) function, and a predicted mannose-specific adhesin (*msa*) were upregulated upon Tween 80 supplementation.

### Effect of Tween on growth parameters

**Fig. 1:**
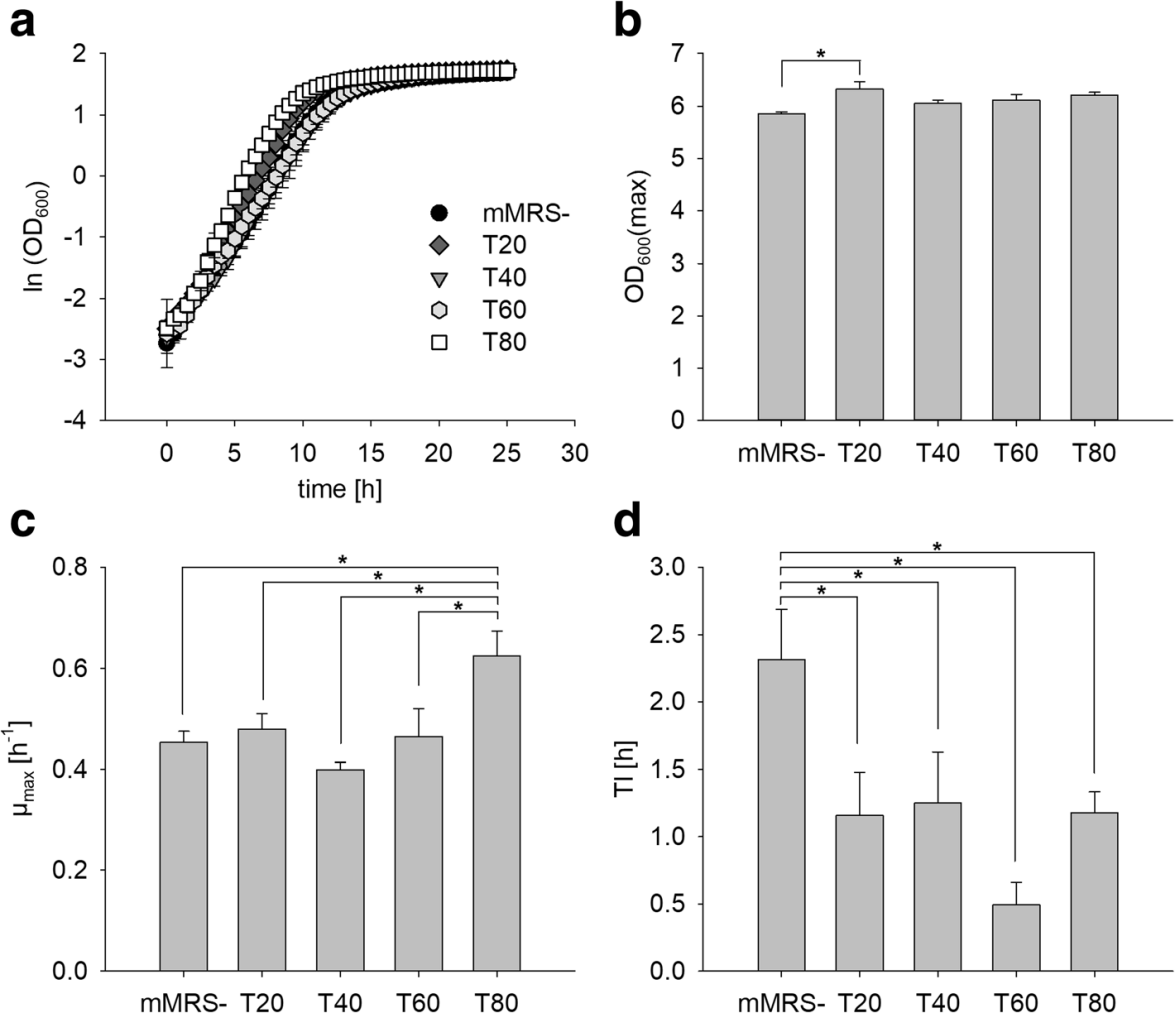
Growth curves and parameters of TMW 1.708 grown in mMRS supplemented with different Tween types. Cells from a 24 h pre-culture in mMRS without Tween supplement (mMRS-) were transferred (1% *v*/v) in fresh mMRS- or mMRS containing 1  g L^− 1^ of either Tween 20 (mMRST20), Tween 40 (mMRST40), Tween 60 (mMRST60), or Tween 80 (mMRST80) and grown at 30 °C for 30 h. **a** Growth curves, **b** maximum OD_600_, **c** maximum growth rate μ_max_ and **d** duration of lag phase Tl are depicted. The presented values are the means of at least three replicates. Error bars represent the standard deviation. Asterisks (*) mark data with statistically significant difference (*p* < 0.05)

Growth curves of *L. plantarum* TMW 1.708 in mMRS- or mMRS supplemented with different Tween types, and the growth parameters derived from these are presented in Fig. 1. Maximum OD_600_ values were only marginally affected regardless of the Tween type added (highest maximum OD_600_ values obtained with Tween 20 and Tween 80, Fig. 1b). The maximum growth rate μ_max_ significantly increased only in the presence of Tween 80 compared to the control without Tween (Fig. 1c). In contrast, all Tween types caused a shortening of the lag time, with Tween 60 having the strongest effect (Fig. 1d). However, lag phase was very short for all samples, which was likely due to pre-cultivation in mMRS- for 24 h, i.e., a pre-culture in the early stationary phase resulting in only minor need for the adaptation of cell metabolism.

### Cellular fatty acid profile

**Fig. 2:**
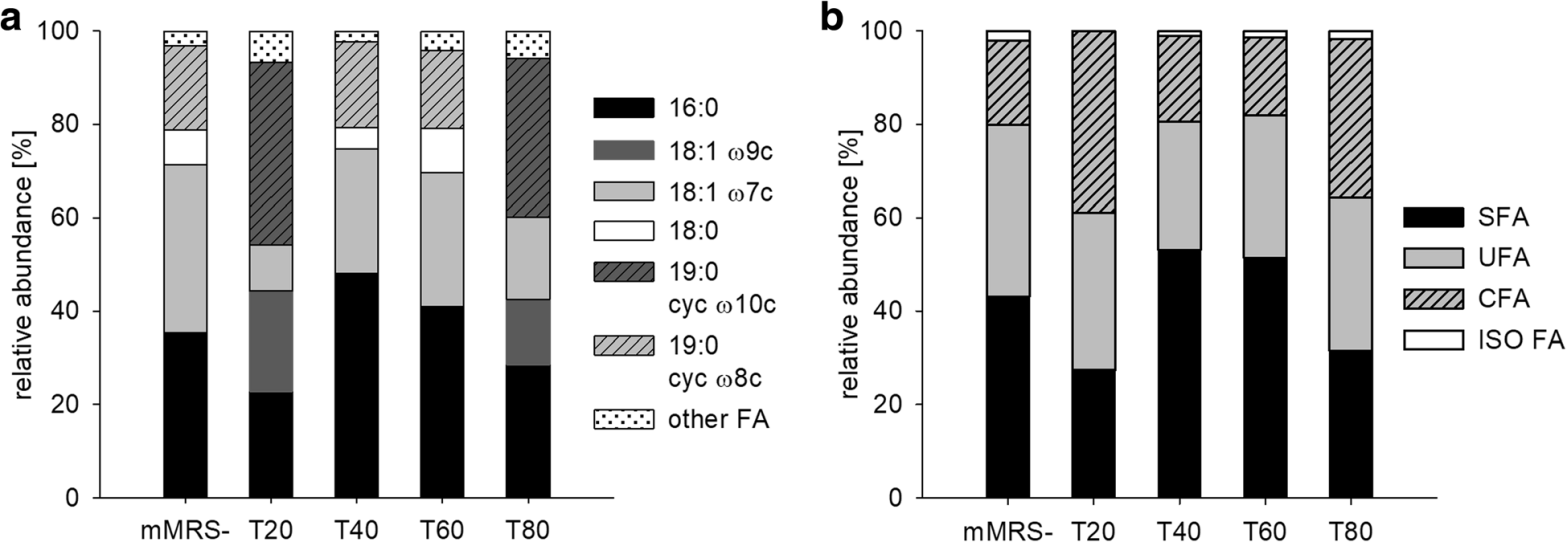
Cellular fatty acid profiles after growth in the presence of different Tween types. Cellular fatty acid profiles were determined after 24 h growth in mMRS-, mMRST20, mMRST40, mMRST60 or mMRST80. **a** Relative abundance of specific fatty acids. Fatty acids with relative abundance lower than 3% are grouped and displayed as “other FA”. **b** Relative abundance of saturated (SFA), unsaturated (UFA), cyclic (CFA) and iso-fatty acids (ISO FA)

We determined the cellular fatty acid profile of *L. plantarum* TMW 1.708 after growth to stationary phase in mMRS medium supplemented with different Tween types. Results are depicted in Fig. 2, expressed as relative abundance of major fatty acids (FA) found (a) and relative abundance of fatty acid types (b), i.e. saturated fatty acids (SFA), unsaturated fatty acids (UFA), cyclic fatty acids (CFA) and branched-chain fatty acids of the iso series (ISO FA).

In cells grown in mMRS-, palmitic (16:0), cis-vaccenic (18:1 ω7cis), stearic (18:0) and lactobacillic acid (19:0 cyclo ω8cis) accounted for > 95% of the total FA content. The addition of Tween 40 and Tween 60 caused a slight increase in the proportions of the exogenous FA palmitic (16:0) and stearic (18,0) acid, respectively, compared to mMRS-.

In contrast, Tween 80 led to substantial amounts of oleic (18:1 ω9cis) and dihydrosterculic (19:0 cyclo ω10cis) acid at the expense of cis-vaccenic, lactobacillic acid and the SFA, palmitic and stearic acid, which was completely eliminated. Tween 20 caused a fatty acid profile similar to Tween 80 and only marginal levels of its specific fatty acid moiety, lauric acid, were detected.

The comparison of the abundance of fatty acid types (Fig. 2b) indicates that Tween 20 and Tween 80 cause a strong overall increase in CFA at the expense of SFA, while UFA levels remained almost constant. Tween 40 and Tween 60 supplementation led to a slight increase in SFA abundance and decreased UFA levels.

### Effect of Tween on HHP inactivation

**Fig. 3:**
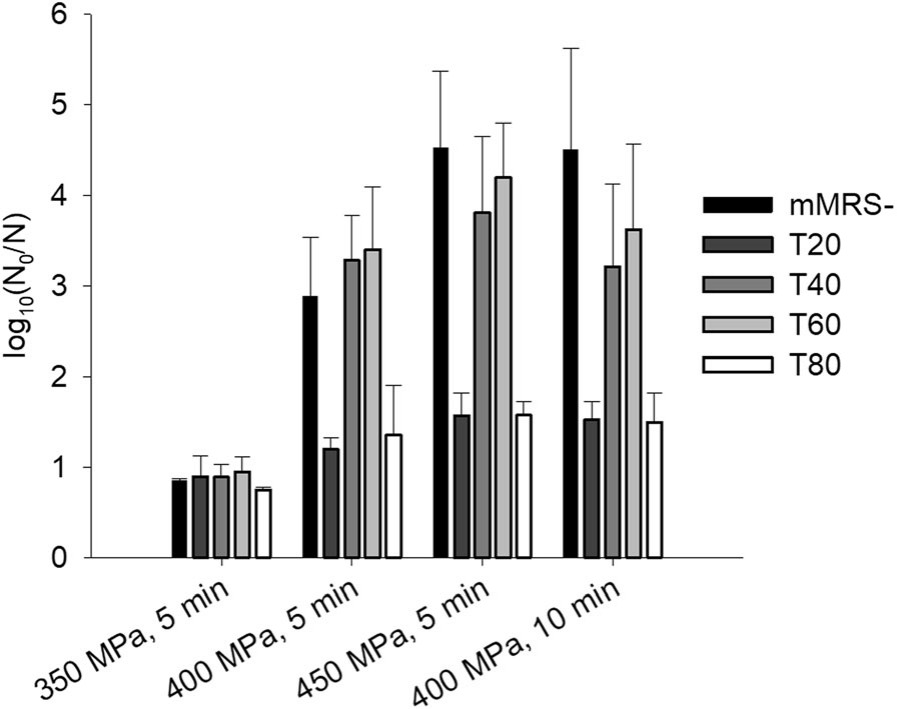
High pressure inactivation of *L. plantarum* cells grown in mMRS medium supplemented with different Tween types. The cells were grown in mMRS- or mMRS supplemented with 1 g L^− 1^ of different Tween types and treated at different pressure/holding time combinations (350/400/450 MPa, 5 min; 400 MPa, 10 min) at 25 °C. Inactivation levels (log reduction of cfu mL^− 1^) were determined on mMRS agar plates. Means and standard deviations derived from at least three independent replicates are depicted

Treatment of TMW 1.708 cells grown in mMRS- or in mMRS containing different Tween types with pressure-time combinations leading to considerable, but not complete inactivation (Fig. 3 and Additional file 2: Table S2) showed that high pressure inactivation levels closely correlate with cellular FA profiles: Medium supplementation with Tween 20 and Tween 80 had a strong protective effect, whereas Tween 40 and 60 led to the development of cells with an HHP tolerance similar to cells grown in mMRS-.

**Fig. 4:**
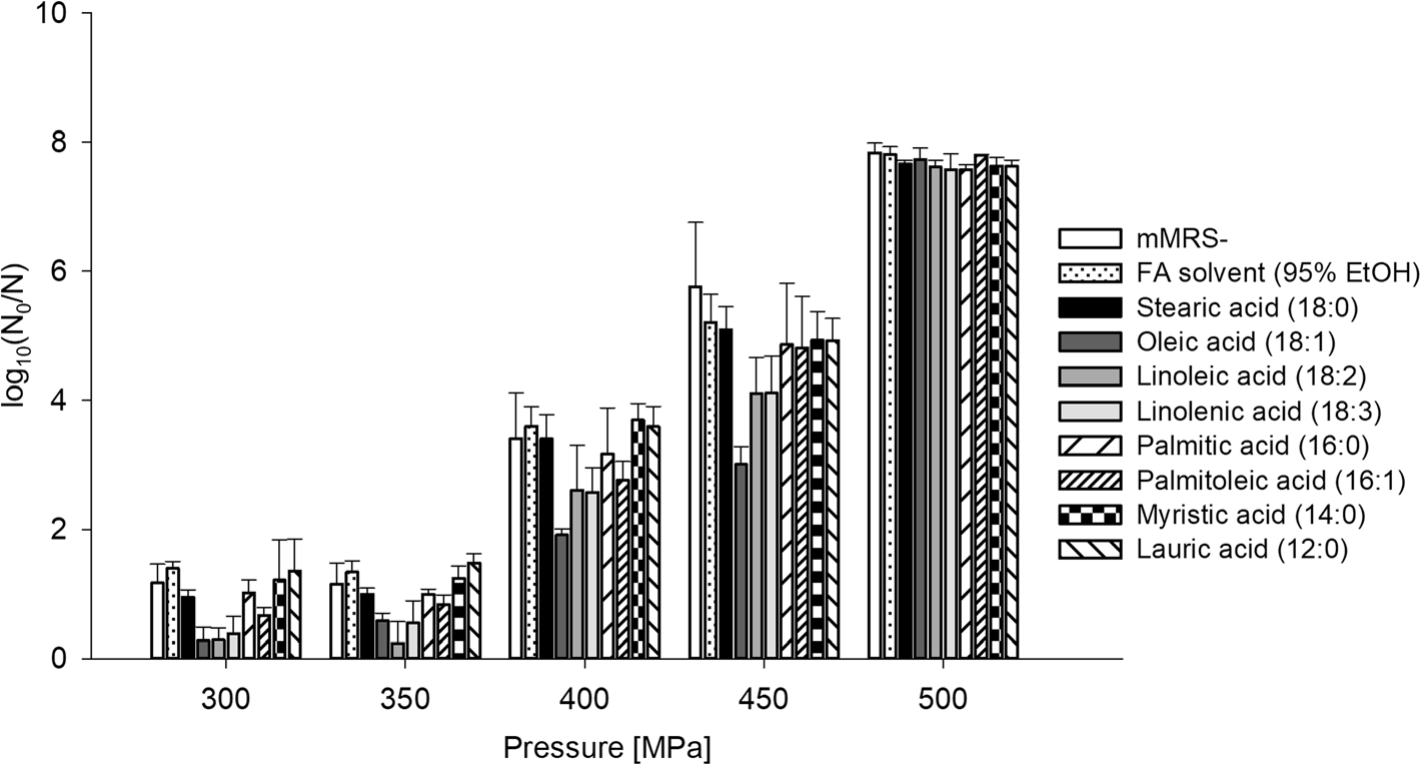
High pressure inactivation of *L. plantarum* after growth in mMRS supplemented with free fatty acids. The cells were grown in mMRS- or mMRS supplemented with 50 mM of different free fatty acids and treated at different pressure levels (300/350/400/450/500 MPa) at 25 °C for 5 min. The log reduction was determined on mMRS agar plates. Shown are the means and standard deviations of at least three independent replicates

HHP inactivation levels of TMW 1.708 grown to stationary phase in mMRS medium supplemented with different free fatty acids are depicted in Fig. 4. Statistically significant differences are summarized in Additional file 3: Table S3. Oleic acid and, to a minor extent, linoleic and linolenic acid were the only FA causing a substantial reduction in inactivation levels. Similar to their corresponding Tween types, Tween 40 and Tween 60, palmitic and stearic acid showed no effect on HHP sensitivity. Unlike Tween 20, lauric acid did not affect HHP sensitivity.

### Effect of Tween 80 on HHP-induced sub-lethal injury

**Fig. 5:**
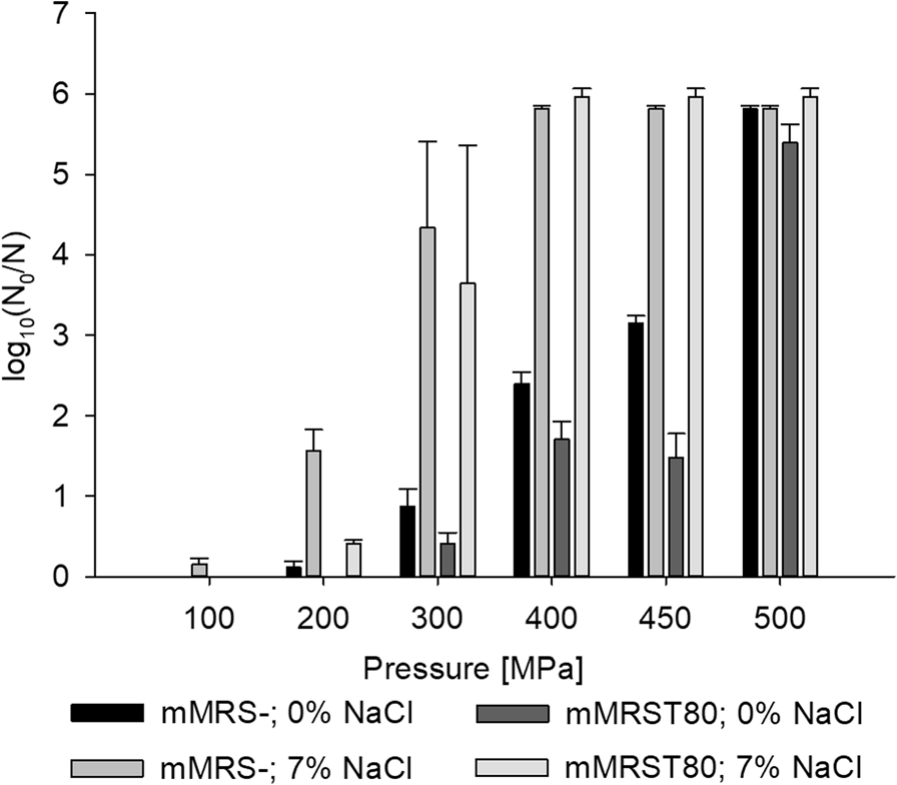
High pressure inactivation of *L. plantarum* after growth in mMRS with or without Tween 80 determined by cultivation on selective and non-selective agar. Cells were grown in mMRS- or mMRST80 and treated at different pressure levels (100/200/300/400/450/500 MPa) at 25 °C for 5 min. The log reduction was determined on mMRS (dark columns) or mMRS-NaCl (light columns) agar plates. Shown are the means and standard deviations of at least three independent replicates

Plating pressure-treated cells in parallel on selective and non-selective agar revealed that at pressure levels between 300 and 450 MPa, where Tween 80 led to a substantial decrease in inactivation levels on non-selective agar, inactivation levels determined on selective agar reached values beyond the detection limit, showing that all surviving cells were sub-lethally injured (see Fig. 5). This demonstrates that Tween 80 either mitigated HHP-induced injury in a large population of cells or increased the ability of cells to overcome HHP-induced injury resulting in a higher total number of cells that are able to recover under favorable conditions.

### Effect of Tween 80 on metabolic activity

**Fig. 6:**
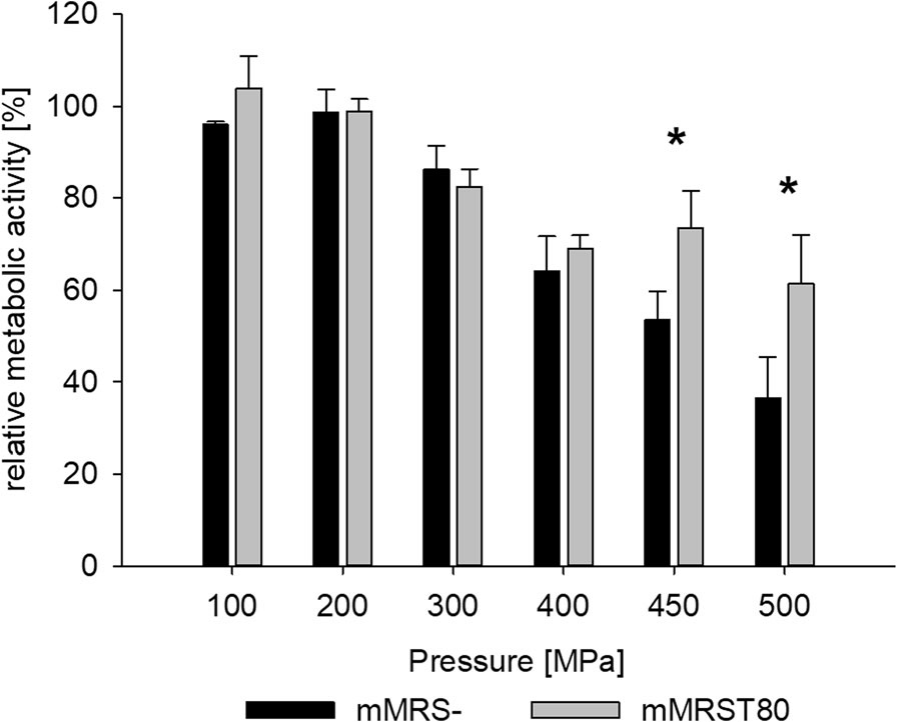
Metabolic activity of *L. plantarum* grown in mMRS- or mMRST80 after treatment at different pressure levels. The cells were grown in mMRS- or mMRS supplemented with 1 g L^− 1^ Tween 80 and treated at different pressure levels (100/200/300/400/450/500) at 25 °C for 5 min. Metabolic activity after pressure treatment was determined using resazurin reduction and compared with the values of untreated cells. Shown are the means and standard deviations of three replicates. Data pairs marked with an asterisk (*) show statistically significant difference (*p* < 0.05)

The metabolic activity of TMW 1.708 grown in the presence or absence of Tween 80 and treated at different pressure levels is shown in Fig. 6. Pressure levels up to 200 MPa did not cause a reduction in metabolic activity, whereas 300 MPa led to a slight reduction after growth in either medium. For pressure levels of 400 MPa and above, metabolic activity decreased rapidly and reached 40% for cells grown in mMRS-, whereas cells grown in mMRST80 retained more than 60% upon treatment at 500 MPa. Thus, the protective effect of Tween 80 also manifests in terms of metabolic activity. These results reveal a large number of viable-but-non-culturable (VBNC) cells at pressure levels of 400 MPa and above, since metabolic activity does not fall below 40 or 60%, although only 1% or less of cells are able to form colonies.

### Effect of Tween 80 on membrane permeabilization under high pressure

#### High pressure-induced protein release

**Fig. 7:**
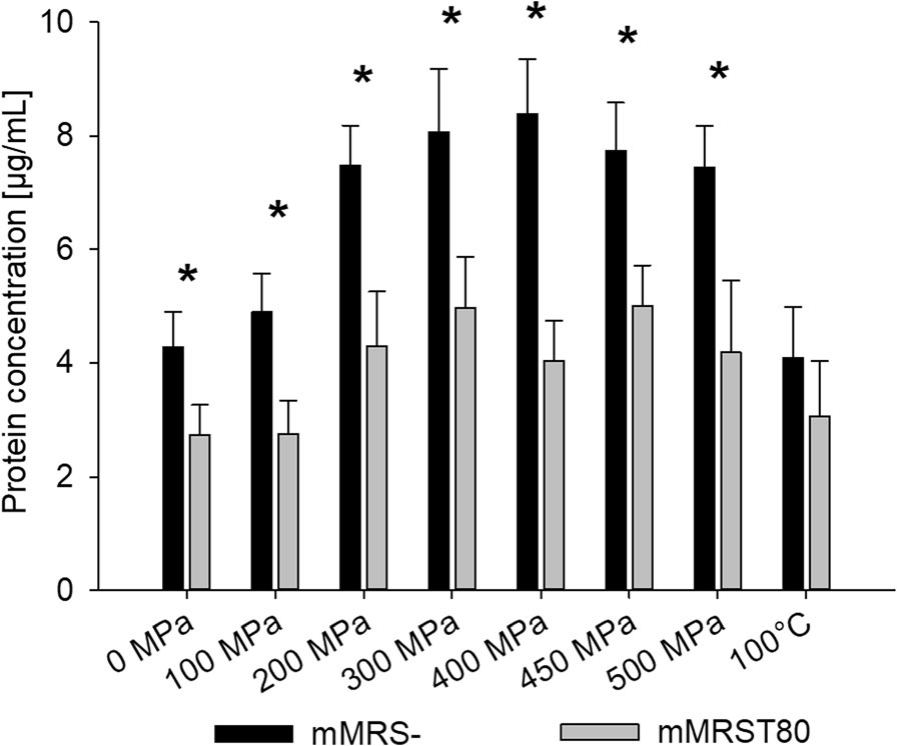
HHP-induced release of proteins by *L. plantarum* cells grown in mMRS- or mMRST80. The cells were grown mMRS medium without and with 1 g L^− 1^ Tween 80 and treated at different pressure levels (100/200/300/400/450/500 MPa) at 25 °C for 5 min or with heat (100 °C, 15 min). The protein concentration in the supernatant after pressure treatment was determined using the Bradford method. Shown are the means and standard deviations of at least seven replicates. Data pairs marked with an asterisk (*) show statistically significant difference (*p* < 0.05)

Protein concentration in the supernatant of cell cultures grown in mMRS- increased from 4 μg mL^− 1^ in untreated samples to 8 μg mL^− 1^ in samples treated at 400 MPa (see Fig. 7). Interestingly, the protein concentration in the supernatant decreased to levels below 8 μg mL^− 1^ after treatments at pressure levels above 400 MPa (see Fig. 7). Cells grown in mMRST80 released less protein at ambient pressure (2.7 μg mL^− 1^), and extracellular protein concentration remained below 5 μg mL^− 1^ after treatment at any pressure, showing that the presence of Tween 80 leads to reduced permeability of the cell membrane under both ambient and high pressure.

#### High pressure-induced uptake of fluorescent dyes

**Fig. 8:**
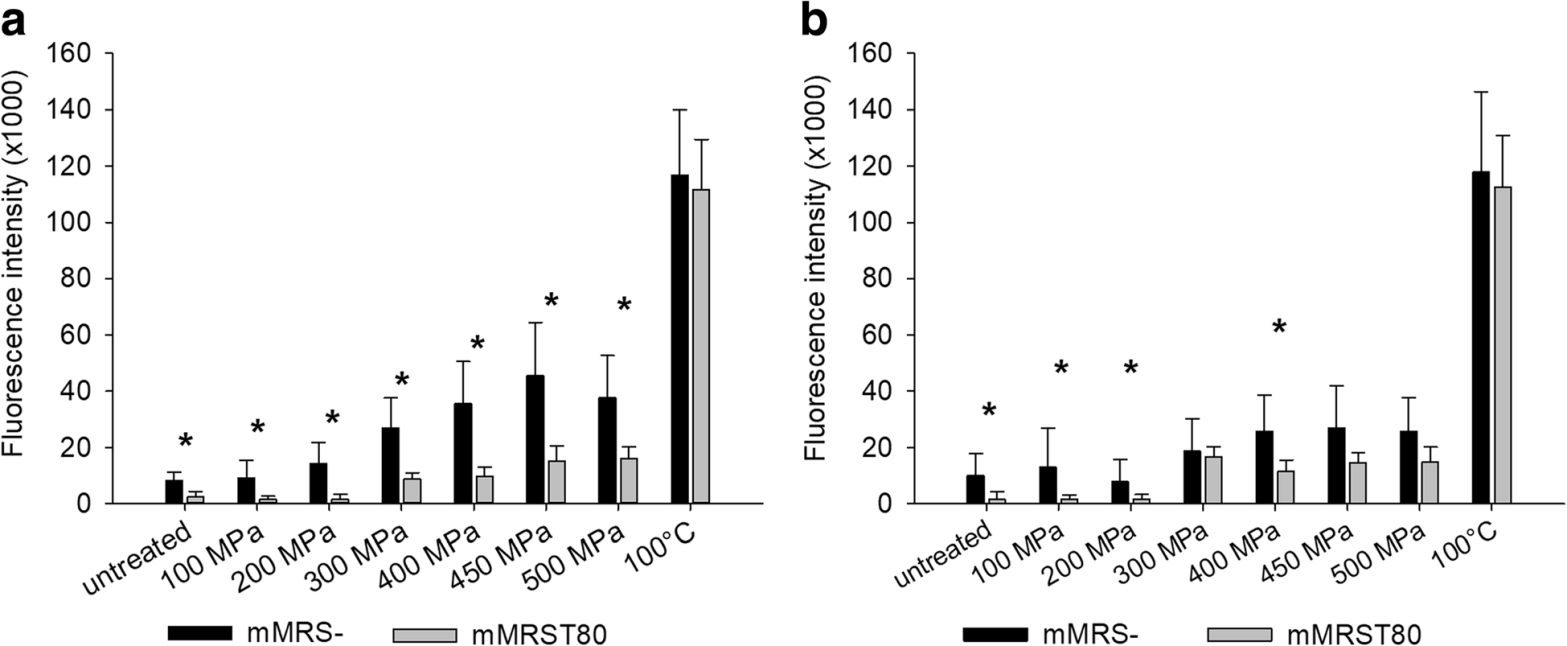
Uptake of PI by *L. plantarum* grown in mMRS- or mMRST80 during and after treatment at different pressure levels. The cells were grown in mMRS without or with 1 g L^−1^ Tween 80 and treated at different pressure levels (100/200/300/400/450/500 MPa) at 25 °C for 5 min or with heat (100 °C, 15 min). The cells were stained with 3 μM PI **a** before or **b** after pressure/heat treatments. Shown are the means and standard deviations of at least five replicates. Data pairs marked with an asterisk (*) show statistically significant difference (p < 0.05)

Cells grown in mMRST80 showed lower initial PI fluorescence than cells grown in mMRS- and the HHP-induced increase in fluorescence was weaker in cells grown in mMRST80 compared with those grown in mMRS-, pointing to reduced transient membrane permeability due to the presence of Tween 80 during growth (Fig. 8a). For permanent membrane permeability, the difference between cells grown in mMRS- and mMRST80 was smaller (Fig. 8b).

Cells grown in mMRS- exhibited lower permanent than transient membrane permeability, showing that a substantial fraction of cells was able to reseal their membranes after pressure release. Growth in mMRST80 led to similar levels of permanent and transient membrane permeability, which indicates that all the HHP-induced membrane damage in these cells was permanent, though generally lower than in cells grown in the absence of Tween 80.

Heat treatment of a cell suspension used in this experiment (10^8^–10^9^ cfu mL^− 1^) at 100 °C for 15 min resulted in no detectable cell growth (data not shown). Taking the detection limit into account, this result means a reduction by at least 6 log, i.e. > 99.9999% of cells unable to recover. HHP treatment at 500 MPa for 5 min led to a reduction in viable cell count by > 5 log cycles (see Fig. 5), meaning that > 99.999% of cells were unable to recover. Since the difference between the fluorescence signals of a sample containing 99.999% and one with 99.9999% fluorescing cells is far below the detection range of the PI assay, similar fluorescence signals of samples treated with HHP (500 MPa, 5 min) and heat (100 °C, 15 min) should have been detected, if all pressure-treated cells unable to recover had permeable cell membranes. However, even with HHP conditions leading to (nearly) complete inactivation (500 MPa) PI fluorescence intensities remained lower than 50% of the maximum achievable signal (heat inactivated positive control), showing that HHP treated cells that lost the ability to recover were still able to partially retain membrane integrity.

Microscopic analysis of PI fluorescence confirmed the results presented above, showing that the whole population of heat-inactivated cells, but only a small fraction of cells pressure-treated at 500 MPa had accumulated PI, although (nearly) the entire pressure-treated population (> 99.999%) had lost the ability to recover (see Fig. 5). In contrast, all cells took up green-fluorescent SYTO®9, which can penetrate both damaged and intact membranes (Additional file 4: Figure S1, Additional file 5: Figure S2, Additional file 6: Figure S3 and Additional file 7: Figure S4). This demonstrates that differences in the PI signal resulted from different numbers of fluorescing cells rather than different fluorescence intensity of the single cells.

**Fig. 9:**
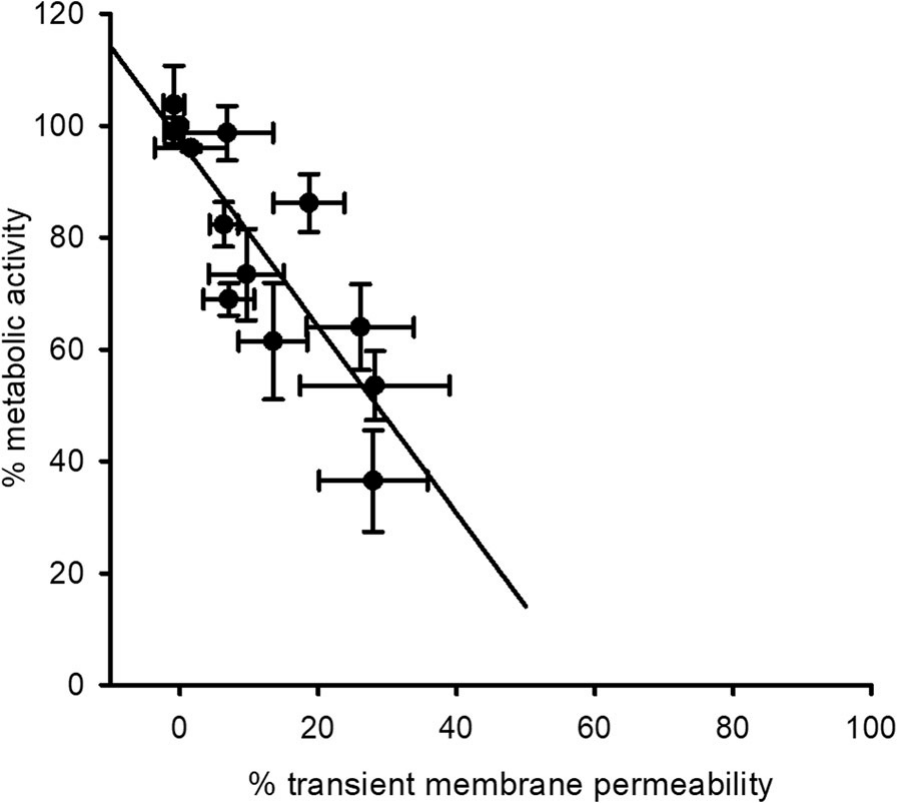
Comparison of metabolic activity and transient membrane permeability after HHP treatments. Relative metabolic activity of *L. plantarum* cells (% of untreated cells) is plotted against their relative transient membrane permeability (% PI fluorescence compared with samples treated at 100 °C, 15 min)

These results clearly show that HHP-induced membrane permeabilization is not necessary to prevent cell growth under favorable conditions (non-selective agar). However, the substantial amount of remaining metabolic activity after HHP treatments shown in Fig. 6 gave rise to the question whether PI uptake (negatively) correlates with the number of VBNC cells. For this, the relative metabolic activity was plotted against the relative transient membrane permeability obtained at various pressure levels (Fig. 9). By increasing the pressure level from 0 to 500 MPa, the metabolic activity of the treated population decreased from 100 to < 40%, whereas membrane permeability not even reached 40% of the maximum reachable value (100% = heat treated positive control). This means that although there seems to exist a linear correlation, metabolic activity is reduced to a stronger extent than membrane integrity by HHP treatment, demonstrating that membrane permeabilization is not required for the loss of metabolic activity under HHP.

## Discussion

We assessed the transcriptomic response of *L. plantarum* strain TMW 1.708 to growth medium supplementation with Tween 80 and observed a significant downregulation of genes involved in the fatty acid biosynthesis pathway. This corroborates a study on *L. casei* [13] that reported the downregulation of fatty acid synthesis genes in the presence of Tween 80, and reflects the reduced need for fatty acid synthesis due to exogenously available oleic acid. The observed downregulation of potassium uptake channels may be explained by the fact that Tween 80 is capable of binding small ions and transporting them through membranes [33], whereas the downregulation of oligopeptide transporter gene *oppC* might result from reduced need for peptide uptake due to the availability of Tween 80 as a carbon source and the concomitant upregulation of amino acid-specific transporters for glutamine (*glnPH2*, *glnQ3*) and lysine (*lysP*) as well as transmembrane transport systems for uncharged ions (*glpF4*) and N-acetylglucosamine (*pts18CBA*). The upregulation of genes involved in the uptake and biosynthesis of pyrimidines and related nucleotides indicates an increased need for nucleic acid components, which may be associated with the growth enhancement by Tween 80 that was observed in this and other studies [6, 34, 35]. At first glance, the upregulation of the transcription repressor of the *pyr* operon, pyrR1, seems contradictory in this context. However, pyrR1 only represses transcription in case of high UMP-to-phosphoribosyl pyrophosphate ratios and, furthermore, possesses minor uracil phosphoribosyltransferase activity, converting uracil and phosphoribosylpyrophosphate to uridine monophosphate (UMP) [32]. In particular, the enhanced activity of the *pyrE* gene, which is involved in the formation of UMP from orothate [31], suggests increased UMP production in the presence of Tween 80. The upregulation of pyrG/CTP synthase, which catalyzes the synthesis of CTP from UTP by utilizing glutamine, enhances the production of CTP. The upregulation of genes coding for a glutamine ABC transporter (glnQ3, glnPH2) and glutamine synthetase (glnA) potentially serves to provide the substrate for CTP synthase. The observed downregulation of glutamate decarboxylase gadB, in turn, might serve to ensure sufficient amounts of glutamate, the substrate of glnA. The upregulation of genes with predicted N-acetyltransferase function, members of the csp4 gene cluster and a predicted mannose-specific adhesion point towards changes in cell surface characteristics the effect of which is currently unclear [36].

In summary, these observations indicate that exogenous oleic acid provided by Tween 80 reduces the need for fatty acid synthesis. Potentially, it is the accompanying energy and material savings that allow for enhanced cell growth, which manifests in the upregulation of genes involved in the synthesis of amino pyrimidine nucleotides needed for de novo assembly of nucleic acids.

In accordance with previous studies, we found that Tween 80 supplementation indeed increases the cellular proportions of oleic acid [6, 9, 12]. Early studies reported the direct incorporation of exogenous FA into cellular lipids of *L. plantarum* [14, 15]. In this context, the nature of the supplied FA appears to be particularly important, since mono-UFA, but not SFA, reduce de novo FA synthesis and enhance growth of lactobacilli [6, 14, 15]. In line with this and in accordance with the results from another study, we found that Tween 80 and Tween 20, but not Tween 40 and Tween 60, are able to enhance growth [6]. The growth-enhancing effect of Tween 80 and Tween 20, which besides lauric acid can contain notable amounts of oleic acid [6, 12], is probably based on the substitution of endogenous cis-vaccenic acid by oleic acid, which only differs in the position of the C-C double bond. We observed this phenomenon by analyzing the cellular fatty acid profiles of *L. plantarum* cells after medium supplementation with different Tween types. Tween 20 and Tween 80 also led to increased total CFA levels at the expense of SFA with the complete elimination of stearic acid. CFA are produced at the onset of stationary phase by CFA synthase, which converts cis-vaccenic and oleic acid into lactobacillic and dihydrosterculic acid, respectively [37, 38]. The substitution of cis-vaccenic acid by oleic acid changes the substrate of CFA synthase. This explains the observed increase in dihydrosterculic acid abundance with concomitantly decreased lactobacillic acid levels.

In contrast, the uptake of lauric acid provided as exogenous SFA in the form of Tween 20, palmitic acid (Tween 40) or stearic acid (Tween 60), was less pronounced, which is in line with results reported previously [14, 15]. However, the reason why exogenous oleic acid, but not SFA enhance growth still needs to be further elucidated.

Since the cell membrane is a target for HHP-mediated inactivation of vegetative cells, and membrane properties have been shown to affect HHP sensitivity of *L. plantarum* [19], we hypothesized that a medium supplementation with different Tween types causing specific alterations in FA profiles can influence HHP sensitivity. Indeed, we found that supplementation with Tween 20, Tween 80, but not Tween 40 or Tween 60, increases the resistance of *L. plantarum* cells to HHP. We further hypothesized that the protective effect of Tween 20 and Tween 80 results from the respective FA coupled to the polyoxyethylene backbone of Tween. To prove this, we examined the HHP sensitivity of TMW 1.708 after growth in mMRS medium supplemented with different free FA and found that only oleic acid and polyunsaturated C18 FA, linoleic and linolenic acid, reduce HHP sensitivity. This shows that the fatty acid part of Tween rather than its hydrophilic poly-oxyethylenesorbitan moiety is responsible for changes in HHP sensitivity. The protective effect of Tween 20 potentially results from traces of oleic acid [6], since other free fatty acids, including lauric acid, did not confer HHP resistance and the fatty acid profile after growth in Tween 20 closely resembled that obtained with Tween 80. Furthermore, we found a correlation between the fatty acid profile and HHP sensitivity, with the characteristic profiles obtained after supplementation with Tween 20 and Tween 80 leading to increased HHP resistance compared to the other conditions. This strongly indicates that HHP sensitivity depends on the fatty acid composition of the cell membrane, which is well-known as a target structure for HHP.

The slightly protective effect observed with the polyunsaturated C18 FA, linoleic and linolenic acid, possibly results from conversion into oleic acid by saturation. According to the available genome data, *L. plantarum* TMW 1.708 possesses the four genes required for this process [39] and there is no evidence for poly-UFA to protect bacteria from HHP inactivation. In contrast, they have been reported to even inhibit the growth of lactobacilli [40].

In order to gain deeper insight into the mechanism underlying the protective effect of Tween 80 against HHP inactivation, we determined the amount of sub-lethal injury by plating pressure-treated TMW 1.708 cells on normal mMRS agar and in parallel on mMRS agar supplemented with the maximum non-inhibitory NaCl concentration. The determination of survival on non-selective agar revealed a strongly protective effect of Tween 80 against inactivation at 400 and 450 MPa. At these pressure levels, the whole surviving population was sub-lethally injured, as detected by plate counts on selective agar, where only undamaged cells can grow. Hence, the differences in inactivation between cells grown in mMRS- and mMRST80 are based on the extent of injury, i.e. whether the damage, which all cells have experienced, is sub-lethal or lethal.

While the plate count method detects cells able to grow and form colonies, some non-dividing cells may still be alive. In order to detect these viable-but-non-culturable (VBNC) cells, we assessed the metabolic activity after HHP treatment using the resazurin reduction assay and found that, despite a substantial inactivation of around two log cycles (i.e. ca. 1% of cells were able to grow out) on non-selective agar after exposure to 400 MPa, cells retained more than 60% of their initial metabolic activity. The discrepancy was even larger at higher pressure levels, where colony counts below (mMRS-) or near (mMRST80) the detection limit (around 6 log cycles) were accompanied by a metabolic activity of around 40% or 60%, respectively. This demonstrates that, after HHP treatment, a large portion of cells is still viable but not culturable under the conditions offered. The fact that the viable fraction was even larger in cell populations grown in the presence of Tween 80 indicates that Tween 80 protects the functionality of crucial redox enzymes. The exact mechanism and, whether the characteristic cellular fatty acid profile plays a role in this context, is still to be elucidated.

High pressure has been shown to damage the bacterial cell membrane, causing increased permeability that manifests in a loss of intracellular material and uptake of extracellular substances [29, 41]. While pressure-induced inactivation of *E. coli* is closely related to membrane permeability [29, 42], the role of membrane damage is not yet clear in gram-positive bacteria [43–45]. However, the fact that (i) HHP can cause alterations in the phase state and permeabilization of biological membranes, (ii) membrane properties have been shown to strongly influence the pressure-induced inactivation of membrane-bound enzymes, which is critical for the survival of *L. plantarum* under HHP [19, 41], and (iii) the a correlation was observed between fatty acid composition and HHP sensitivity supported our hypothesis that the cell membrane plays a key role in Tween 80-mediated HHP resistance. We therefore determined pressure-induced membrane damage via measuring the HHP-mediated release of protein and uptake of propidium iodide (PI). Our results show that growth in the presence of Tween 80 reduces protein release from untreated cells, indicating lower membrane permeability in general, and substantially diminishes pressure-induced protein release, pointing towards increased pressure-stability of the membrane. The amount of released protein increased only up to a specific pressure level (400 MPa), whereas higher pressures and heat treatment caused lower protein release. A similar effect was observed in studies on *E. coli* [29, 42], and it was speculated whether it results from (i) the formation of intracellular aggregates of denatured proteins, which are unable to penetrate the peptidoglycan network, and/or (ii) structural changes of the cell envelope, which prevent proteins from escaping the cell [29, 46].

Despite their inability to grow on non-selective agar, only a minor fraction of cells treated at pressure levels of 400 MPa or higher exhibited membranes permeable to PI. These results, which are in line with previous studies on lactobacilli [41, 43, 44], and the fact that HHP impaired metabolic activity much more than membrane integrity, leads to the conclusion that membrane permeabilization per se is not the main cause for pressure-induced inactivation of *L. plantarum*. Moreover, neither metabolic activity nor membrane integrity was impaired to an extent that would explain the great loss of viability detected on non-selective agar. A possible explanation might be found in the data of Govers et al. who showed in *E. coli* that HHP-induced disassembly of aggregates of misfolded proteins impedes resuscitation until these proteins are re-assembled to proper inclusion bodies, although the cells are viable and metabolically active [47]. Possibly, the *L. plantarum* cells used here behaved in a similar manner, with many cells with intact membranes and active metabolism being unable to resume proliferation because they failed to reassemble their protein aggregates.

Even though the HHP-induced loss of membrane integrity does not account for the observed failure to grow on non-selective agar, the reduced basic and pressure-induced membrane permeability in cells grown in the presence of Tween 80 compared to mMRS- cannot be denied. Furthermore, the question remains, how Tween 80, and possibly the associated fatty acid profile, confer pressure resistance.

Both the increased survival rate and the reduced membrane permeability of cells grown in the presence of Tween 80 might be the result of the elevated CFA content of these cells. Like most other bacteria, lactobacilli use CFA synthase to convert monounsaturated fatty acids into the corresponding CFA from the beginning of stationary growth phase on [38, 48]. Furthermore, CFA have been linked to the resistance of lactobacilli to various stress factors, including high acidity [10, 49], bile acids [50], cold stress [51] and high metabolite concentrations in stationary growth phase [48, 52]. Moreover, CFA have been shown to confer high pressure resistance to *E. coli*, and CFA synthase knockout mutants of *E. coli* showed stronger membrane permeabilization and loss of viability under HHP than their wild type counterparts [42, 53].

According to a recent study [54], cis-CFA, such as lactobacillic and dihydrosterculic acids, have a dual effect on membrane properties: on the one hand, they increase overall fluidity and diffusion compared to SFA and UFA. This leads to a shift of the phase transition from liquid-crystalline to gel phase towards higher pressure levels. Thus, important membrane-bound enzymes are protected and membrane functionality is ensured up to higher pressure levels, resulting in higher survival rates, as observed after growth in the presence of Tween 80. On the other hand, CFA show a higher degree of order within the acyl chain than the analogous UFA. The higher rigidity of the hydrocarbon chains results in reduced permeability to small molecules, such as many toxic compounds and dyes like PI. This could explain the reduced permeability to PI in cells grown in the presence of Tween 80.

Furthermore, CFA might increase cell viability because they are inert to oxidation by reactive oxygen species produced under high pressure. Methylenation of unsaturated FA (i.e. formation of CFA) has been hypothesized to improve resistance to superoxide, singlet oxygen, ozonolysis and oxidative stress in general [48].

## Conclusion

Transcriptomic analysis suggest that the growth-enhancing effect of Tween 80 is based primarily on energy and resource savings due to the downregulation of de novo fatty acid synthesis. While the effect of Tween 80 on membrane fatty acid composition of lactobacilli and the importance of the membrane for HHP tolerance have been described earlier, we could, for the first time, directly link Tween 80 and free oleic acid to increased HHP tolerance. Our results further show that Tween 80 diminishes the pressure-induced loss of metabolic activity, protein release and membrane permeabilization whereas membrane damage is not a prerequisite for loss viability or metabolic activity.

## Additional files

Additional file 1: **Table S1.** Differentially expressed genes upon the addition of Tween 80 to the growth medium. (DOCX 22 kb)

Additional file 2: **Table S2.** Statistical analysis of HHP inactivation rates of L. plantarum TMW 1.708 after supplementation with different Tween types. (DOCX 14 kb)

Additional file 3: **Table S3.** Statistical analysis of HHP inactivation rates of L. plantarum TMW 1.708 after supplementation with different free fatty acids. (DOCX 16 kb)

Additional file 4: **Figure S1.** L. plantarum grown in mMRS- and stained with PI before HHP treatment or heat inactivation. (DOCX 286 kb)

Additional file 5: **Figure S2.** L. plantarum grown in mMRST80 and stained with PI before HHP treatment or heat inactivation. (DOCX 218 kb)

Additional file 6: **Figure S3.** L. plantarum grown in mMRS- and stained with PI after HHP treatment or heat inactivation. (DOCX 176 kb)

Additional file 7: **Figure S4.** L. plantarum grown in mMRST80 and stained with PI after HHP treatment or heat inactivation. (DOCX 217 kb)
