# Supplementary material for: Interrelation between Tween and the membrane properties and high pressure tolerance of Lactobacillus plantarum
Source: BMC Microbiol. 2018 Jul 13;18:72. doi: 10.1186/s12866-018-1203-y (PMC6044075; doi:10.1186/s12866-018-1203-y)
Supplement: Supplementary file 1 — Table S1. Differentially expressed genes upon the addition of Tween 80 to the growth medium. (DOCX 22 kb) [file 12866_2018_1203_MOESM1_ESM.docx]

**Additional file**

Additional file 1: Table S1: Differentially expressed genes upon the addition of Tween 80 to the growth medium. A)

| **sseqid** | **Name** | **Product/Function** | **General functional category** | **log2 expr. change  (mMRST80 vs. mMRS-)** |
| --- | --- | --- | --- | --- |
| BIZ33_13110 |  | Fatty acid-binding protein (DegV family) | Fatty acid metabolism | -6.43 |
|  |  | part of BIZ33_13110 | Fatty acid metabolism | -6.25 |
| BIZ33_06635 | fabZ | 3-hydroxyacyl-[acyl-carrier-protein] dehydratase FabZ (EC 4.2.1.59) | Fatty acid biosynthesis, Biotin metabolism | -4.95 |
| BIZ33_06640 | fabH | 3-Oxoacyl-ACP synthase (EC 2.3.1.180) | Fatty acid biosynthesis | -4.80 |
| BIZ33_06645 | acpA2 | Acyl carrier protein | Fatty acid biosynthesis | -4.72 |
| BIZ33_06650 | fabD | ACP S-malonyltransferase (EC 2.3.1.39) | Fatty acid biosynthesis | -4.50 |
| BIZ33_06655 | fabG1 | beta-ketoacyl-ACP reductase (EC 1.1.1.100) | Fatty acid biosynthesis | -4.38 |
| BIZ33_06660 | fabF | beta-ketoacyl-[acyl-carrier-protein] synthase II (EC 2.3.1.179) | Fatty acid biosynthesis | -4.14 |
| BIZ33_06665 | accB2 | Acetyl-CoA carboxylase biotin carboxyl carrier protein subunit | Fatty acid biosynthesis | -4.04 |
| BIZ33_06670 | fabZ2 | beta-hydroxyacyl-ACP dehydratase (EC 4.2.1.59) | Fatty acid biosynthesis | -3.99 |
| BIZ33_06675 | accC2 | Acetyl-CoA carboxylase biotin carboxylase subunit (EC 6.3.4.14) | Fatty acid biosynthesis | -3.83 |
| BIZ33_06680 | accD2 | Acetyl-CoA carboxylase subunit beta (EC 6.4.1.2) | Fatty acid biosynthesis | -3.62 |
| BIZ33_06685 | accA | Acetyl-CoA carboxylase carboxyl transferase subunit alpha (EC 6.4.1.2) | Fatty acid biosynthesis | -3.55 |
| BIZ33_06690 | fabI | Enoyl-[acyl-carrier-protein] reductase (EC 1.3.1.9) | Fatty acid biosynthesis | -3.36 |
|  |  | part of BIZ33_06695 |  | -3.28 |
| BIZ33_06695 |  | Phosphopantetheinyl transferase | Fatty acid biosynthesis | -3.22 |
| BIZ33_06620 |  | Uncharacterized membrane protein | - | -2.75 |
| BIZ33_13555 |  | Uncharacterized protein | - | -2.69 |
| BIZ33_02005 | kup1 | Kup system potassium uptake protein | Potassium uptake | -2.62 |
| BIZ33_13550 |  | Membrane-bound cell surface alpha-beta hydrolase | - | -2.58 |
| BIZ33_10730 |  | Glycosyl transferase (family 1) | - | -1.94 |
| BIZ33_13185 | kup2 | Kup system potassium uptake protein | Potassium uptake | -1.94 |
| BIZ33_13665 |  | Glutamate decarboxylase (EC 4.1.1.15) | Amino acid transport and metabolism | -1.92 |
| BIZ33_06705 |  | Transcriptional regulator (LysR family) | transcription regulation | -1.85 |
| BIZ33_05085 | oppC | Transmembrane oligopeptide ABC transporter (TC 3.A.1.5.1) | Amino acid transport and metabolism | -1.80 |
| BIZ33_12155 |  | Extracellular transglycosylase with LysM peptidoglycan binding domain | - | -1.79 |

B)

| **sseqid** | **Name** | **Product/Function** | **General functional category** | **log2 expr. change (mMRST80 vs. mMRS-)** |
| --- | --- | --- | --- | --- |
| BIZ33_03065 | pstB1 | Phosphate ABC transporter ATP-binding protein (TC 3.A.1.7.1) | phosphate transport | 1.20 |
| BIZ33_12070 |  | nucleotide binding protein, universal stress protein UspA |  | 1.23 |
| BIZ33_08595 | cps4J | Polysaccharide repeat unit transporter (Flippase) | polysacchride synthesis | 1.23 |
| BIZ33_08605 | cps4H | Polysaccharide polymerase | polysacchride synthesis | 1.24 |
| BIZ33_12290 |  | Amino acid transport protein | Amino acid transport and metabolism | 1.25 |
| BIZ33_10280 | pts18CBA | PTS N-acetylglucosamine transporter subunit IIABC (EC 2.7.1.69) | Amino sugar and nucleotide sugar metabolism; Phosphotransferase system (PTS) | 1.26 |
| BIZ33_12780 |  | hypothetical protein |  | 1.26 |
| BIZ33_08610 | cps4G | Glycosyl transferase (family 1) |  | 1.30 |
| BIZ33_05610 |  | N-acetyltransferase (GNAT family) |  | 1.40 |
| - | mntH3 | Manganese transport protein | Manganese uptake | 1.41 |
| BIZ33_10925 |  | Xanthine permease | Xanthine/uracil uptake | 1.42 |
| BIZ33_06270 |  | Transcriptional regulator (MerR family) | Transcription regulation/Amino acid metabolism | 1.43 |
| BIZ33_08600 | cps4I | Glycosyltransferase (family 2) |  | 1.46 |
| - |  | hypothetical protein |  | 1.47 |
| BIZ33_06275 | glnA | Glutamine synthetase (type I) (EC 6.3.1.2) | Amino acid transport and metabolism | 1.50 |
| BIZ33_10915 | pyrR1 | Pyrimidine nucleotide operon transcriptional regulator/Uracil phosphoribosyltransferase (EC 2.4.2.9) | pyrimidine metabolism | 1.53 |
| BIZ33_04935 |  | Transcriptional regulator (MarR family) | mannose adhesion | 1.55 |
| - | msa | Mannose-specific adhesin, LPXTG-motif cell wall anchor | mannose adhesion | 1.58 |
| BIZ33_04925 | glpF4 | Glycerol uptake facilitator protein | Glycerol uptake/metabolism | 1.74 |
| BIZ33_13095 |  | N-acetyltransferase (GNAT family) | - | 1.79 |
| BIZ33_10880 | pyrE | Orotate phosphoribosyltransferase (EC 2.4.2.10) | Pyrimidine metabolism | 1.81 |
| BIZ33_09855 | pyrP | Uracil permease | Pyrimidine metabolism | 1.90 |
| BIZ33_01905 | pyrG | CTP synthase (EC 6.3.4.2) | Pyrimidine metabolism | 1.94 |
| BIZ33_04195 | lysP | Lysine-specific permease | Amino acid transport and metabolism | 2.07 |
| BIZ33_08650 | glnQ3 | Glutamine ABC transporter ATP-binding protein | Amino acid transport and metabolism | 2.73 |
| BIZ33_08655 | glnPH2 | Glutamine ABC transporter substrate binding and permease protein | Amino acid transport and metabolism | 2.81 |
