# Supplementary material for: Interrelation between Tween and the membrane properties and high pressure tolerance of Lactobacillus plantarum
Source: BMC Microbiol. 2018 Jul 13;18:72. doi: 10.1186/s12866-018-1203-y (PMC6044075; doi:10.1186/s12866-018-1203-y)
Supplement: Supplementary file 2 — Table S2. Statistical analysis of HHP inactivation rates of L. plantarum TMW 1.708 after supplementation with different Tween types. (DOCX 14 kb) [file 12866_2018_1203_MOESM2_ESM.docx]

**Additional file**

Additional file 2: Table S2 Statistical analysis of HHP inactivation rates of L. plantarum TMW 1.708 after supplementation with different Tween types.

|  | 350 MPa, 5 min | 400 MPa, 5 min | 450 MPa, 5 min | 400 MPa, 10 min |
| --- | --- | --- | --- | --- |
| mMRS- | - | 2, 5 | 2, 5 | 2, 5 |
| Tween 20 | - | 1, 3, 4 | 1, 3, 4 | 1, 4 |
| Tween 40 | - | 2, 5 | 2, 5 | 2, 5 |
| Tween 60 | - | 2, 5 | 2, 5 | 2, 5 |
| Tween 80 | - | 1, 3, 4 | 1, 3, 4 | 1, 4 |
