# Supplementary material for: Interrelation between Tween and the membrane properties and high pressure tolerance of Lactobacillus plantarum
Source: BMC Microbiol. 2018 Jul 13;18:72. doi: 10.1186/s12866-018-1203-y (PMC6044075; doi:10.1186/s12866-018-1203-y)
Supplement: Supplementary file 3 — Table S3. Statistical analysis of HHP inactivation rates of L. plantarum TMW 1.708 after supplementation with different free fatty acids. (DOCX 16 kb) [file 12866_2018_1203_MOESM3_ESM.docx]

Additional file

Additional file 3: Table S3: Statistical analysis of HHP inactivation rates of L. plantarum TMW 1.708 after supplementation with different free fatty acids. HHP-induced log reductions values were compared by analysis of variance.

|  | 300 | 350 | 400 | 450 | 500 |
| --- | --- | --- | --- | --- | --- |
| mMRS- | 4, 5, 6 | 4, 5, 6 | 4 | 4 | - |
| FA solvent | 4, 5, 6 | 4, 5, 6 | 4 | 4 | - |
| Stearic acid | - | 5 | 4 | 4 | - |
| Oleic acid | 1, 2, 9, 10 | 1, 2, 8, 9, 10 | 1, 2, 3, 7, 8, 9, 10 | 1, 2, 3, 7, 8, 9, 10 | - |
| Linoleic acid | 1, 2, 9, 10 | 1, 2, 3, 7, 9, 10 | - | - | - |
| Linolenic acid | 1, 2, 9, 10 | 1, 2, 9, 10 | - | - | - |
| Palmitic acid | - | 5 | 4 | 4 | - |
| Palmitoleic acid | - | 4, 10 | 4 | 4 | - |
| Myristic acid | 4, 5, 6 | 4, 5, 6 | 4 | 4 | - |
| Lauric acid | 4, 5, 6 | 4, 5, 6 | 4 | 4 | - |
