# Supplementary material for: Interrelation between Tween and the membrane properties and high pressure tolerance of Lactobacillus plantarum
Source: BMC Microbiol. 2018 Jul 13;18:72. doi: 10.1186/s12866-018-1203-y (PMC6044075; doi:10.1186/s12866-018-1203-y)
Supplement: Supplementary file 6 — Figure S3. L. plantarum grown in mMRS- and stained with PI after HHP treatment or heat inactivation. (DOCX 176 kb) [file 12866_2018_1203_MOESM6_ESM.docx]

Additional file

Additional file 6: Figure S3: Growth in mMRS-, staining after treatment

|  | SYTO^®^9 | PI |
| --- | --- | --- |
| No treatment | 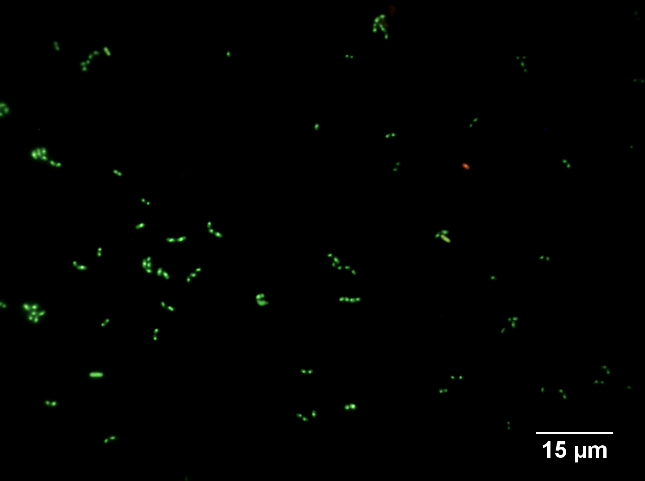 | 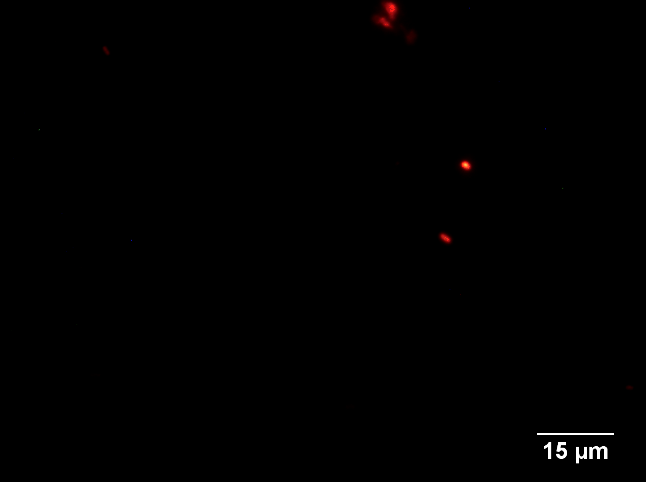 |
| 500 MPa, 25 °C, 5 min | 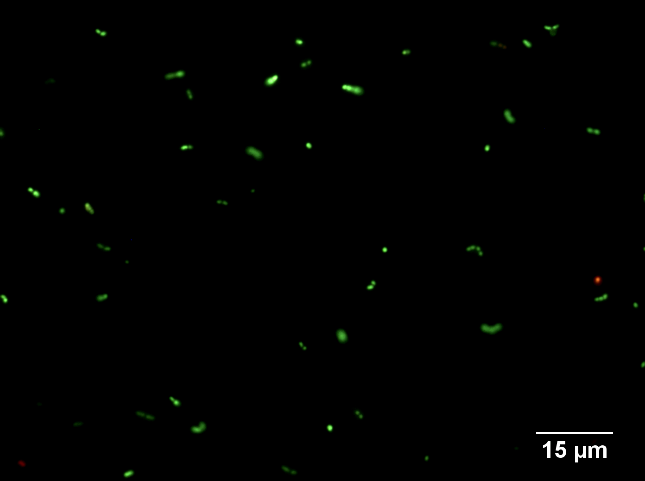 | 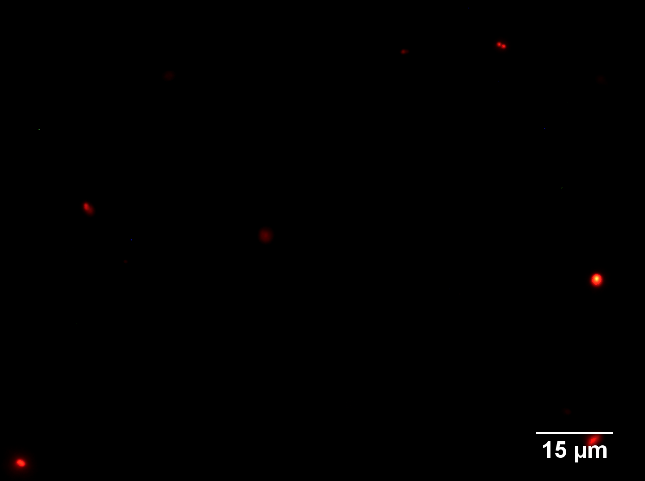 |
| 0.1 MPa, 100 °C, 15 min | 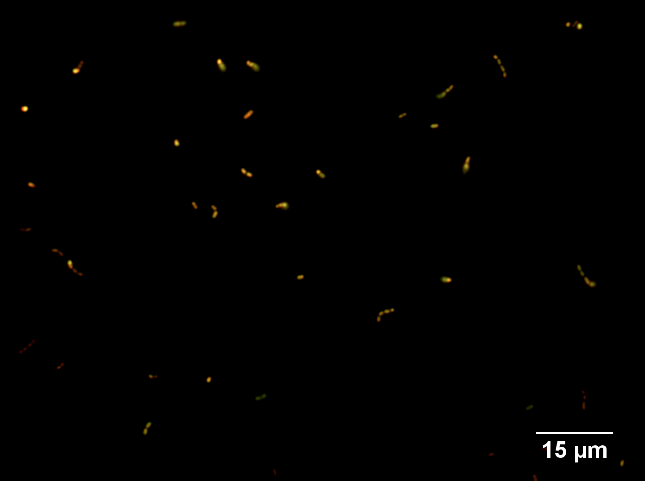 | 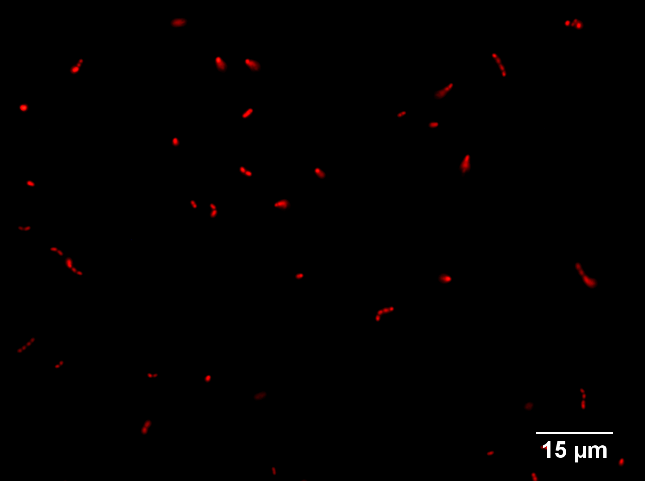 |
